# TCR β CDR3 repertoire signatures in paired PBMC and BALF from children with mycoplasma pneumoniae pneumonia

**DOI:** 10.3389/fimmu.2026.1842653

**Published:** 2026-09-11

**Authors:** Xingzhu Liu, Chuxiong Gong, Tingting Hao, Jun Li, Xing Zhang, Zhongjian Su, Yanfei Chen, Yangfei Yang, Yuqin Wu

**Affiliations:** 1 Department of Special Needs Ward, Kunming Children’s Hospital, Kunming, Yunnan, China; 2 Department of Cardiology, Kunming Children’s Hospital, Kunming, Yunnan, China; 3 Immunology, Center of Immunomolecular Engineering, Innovation and Practice Base for Graduate Students Education, Zunyi Medical University, Zunyi, China

**Keywords:** bronchoalveolar lavage fluid, CDR3 repertoire, mycoplasma pneumoniae, pediatric pneumonia, TCR

## Abstract

**Background:**

Mycoplasma pneumoniae (MP) is a leading cause of community-acquired pneumonia in children, yet the characteristics of the T cell receptor (TCR) repertoire in the local pulmonary environment remain poorly defined.

**Methods:**

Paired PBMC and bronchoalveolar lavage fluid (BALF) samples from four MP-infected children were subjected to TCR β CDR3 repertoire sequencing, followed by analysis of clonotype diversity, repertoire overlap, V/J gene usage, CDR3 length distribution, database annotation and k-mer profiling. The main findings were further validated in an independent cohort including 13 MP patients and 20 healthy children (HC).

**Results:**

Although BALF contained fewer clonotypes than paired PBMC samples, it showed a trend toward a higher proportion of recurrent clonotypes and long (≥16 AA) CDR3 sequences. Only a small fraction of clonotypes were shared between paired PBMC and BALF samples, with minimal inter-individual overlap; however, V and J gene usage patterns were more similar within paired samples than between different individuals. A minority of shared clonotypes matched known antigen-specific sequences, mainly against viruses, and k-mer analysis revealed distinct motif patterns in BALF. In the expanded cohort, repertoire diversity was significantly reduced in both MP_BALF and MP_PBMC compared with HC_PBMC. Several TRBV and TRBJ genes showed differential usage between MP patients and healthy controls, and the proportion of long (≥16 AA) CDR3 sequences was significantly increased only in MP_PBMC. Recurrent clonotypes identified in BALF also showed a trend toward higher frequencies in MP_PBMC than in HC_PBMC.

**Conclusion:**

Pediatric MP pneumonia is associated with distinct compartment-specific TCR β repertoire features, including altered repertoire diversity, biased TRBV/TRBJ gene usage, and compartment-dependent CDR3 characteristics, providing insight into systemic and local adaptive immune responses during MP infection.

## Introduction

Mycoplasma pneumoniae (MP) infection is one of the most common causes of community-acquired pneumonia in children and can induce excessive and dysregulated immune responses in severe cases (1, 2). While innate immunity contributes to initial pathogen recognition, adaptive immune responses, particularly T lymphocytes, play a central role in MP clearance and immunopathology (3–5). Among T cells, the diversity and clonal expansion of T cell receptors (TCRs) determine antigen recognition capacity and reflect ongoing immune activation (6, 7). However, the characteristics of the TCR repertoire in different anatomical compartments during MP infection remain poorly understood.

The β chain of the TCR, especially the complementarity-determining region 3 (CDR3), represents the most variable part of the receptor and is critical for antigen specificity (8). High-throughput sequencing of the TCR β repertoire provides a powerful approach for investigating T cell diversity, clonotype expansion, V(D)J gene usage, and disease-associated immune signatures (9, 10). Previous studies have described alterations in the peripheral immune profile of MP-infected patients (11–13); however, most of them focused primarily on peripheral blood, with limited attention to local immune responses in the lower respiratory tract, such as bronchoalveolar lavage fluid (BALF).

**Figure 1:**
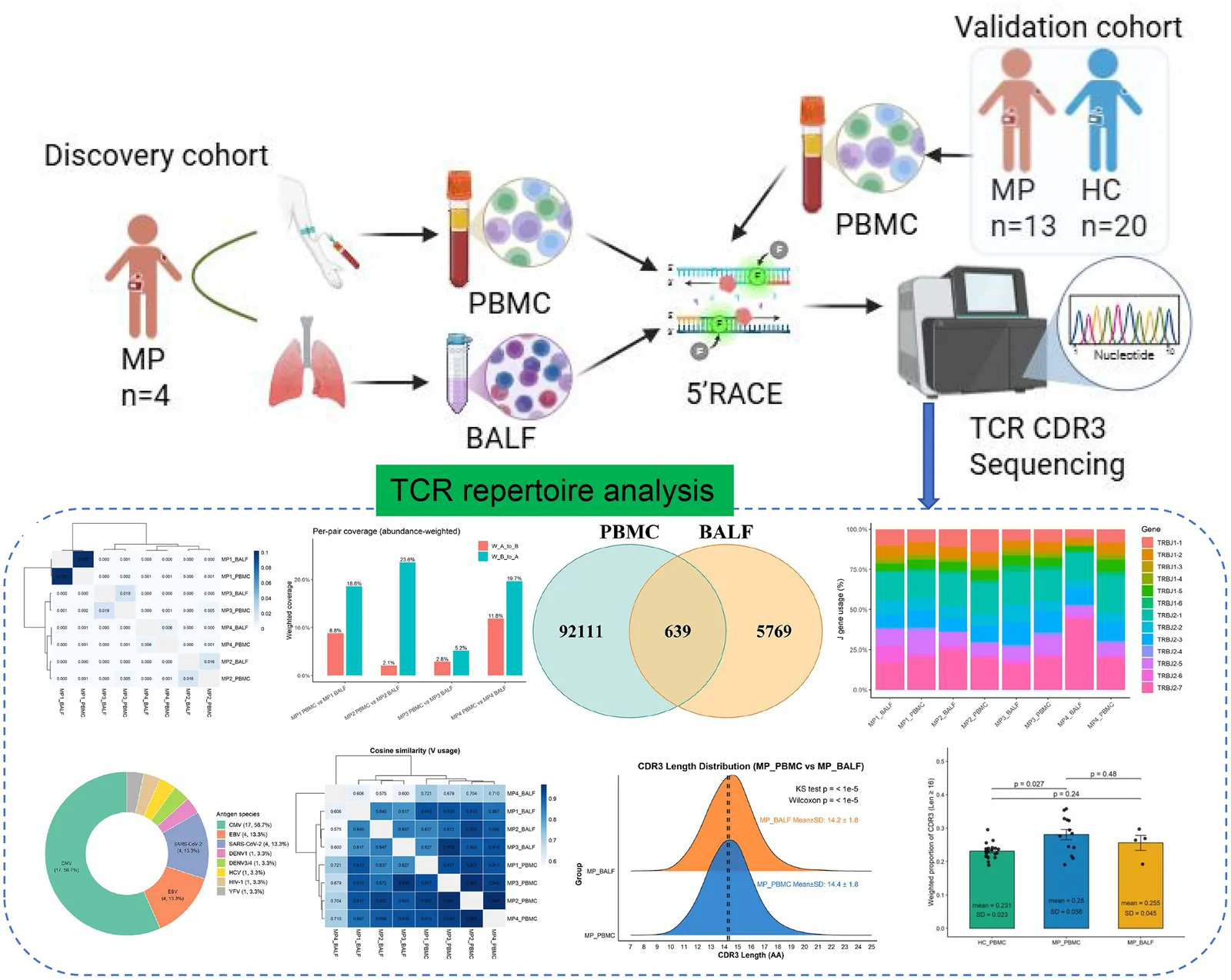
Study design and analytical workflow. The study consisted of two complementary components. First, a paired exploratory analysis was performed using bronchoalveolar lavage fluid (BALF) and peripheral blood mononuclear cell (PBMC) samples collected from four children with Mycoplasma pneumoniae pneumonia (MP) to characterize tissue-specific TCR β repertoire features. Second, an independent PBMC cohort including MP patients and healthy controls (HC) was used for validation analyses. TCR β CDR3 repertoire sequencing was performed for all samples, followed by analyses of repertoire diversity, V(D)J gene usage, clonotype sharing, and validation of selected repertoire features.

In this study, we performed a comprehensive analysis of the productive TCR β repertoires in paired BALF and peripheral blood mononuclear cell (PBMC) samples from children with MP infection, and validated in an independent cohort including 13 MP patients and 20 healthy children (HC) (**Figure 1**). By analyzing CDR3 length distribution, clonal expansion, repertoire diversity, V and J gene usage, clonotype sharing, and motif features, we aimed to characterize both systemic and local T cell immune landscapes in MP-infected children, and to identify potential repertoire signatures associated with disease-related immune responses.

## Materials and methods

### Study population and sample collection

All samples were obtained from children admitted to Kunming Children’s Hospital between March 2024 and August 2025. During this study period, all children with clinically suspected Mycoplasma pneumoniae pneumonia were screened consecutively for eligibility. The study enrolled patients who were clinically diagnosed with Mycoplasma pneumoniae infection. The diagnosis was established based on compatible clinical manifestations together with positive laboratory evidence of Mycoplasma pneumoniae infection (MP-DNA and/or MP-IgM positivity). Patients with severe pulmonary involvement who required bronchoscopy and bronchoalveolar lavage as part of routine clinical management were considered eligible for inclusion. The age of the enrolled children ranged from 7 to 93 months, with an approximately equal distribution of males and females.

The four paired patients (**Supplementary Table 1**) were eligible for inclusion if they had a confirmed diagnosis of Mycoplasma pneumoniae pneumonia according to institutional clinical guidelines and underwent bronchoscopy with bronchoalveolar lavage as part of routine clinical care. Patients were excluded if paired peripheral blood and BALF samples were unavailable, sample volume was insufficient for immune repertoire sequencing, clinical information was incomplete, or sequencing quality-control requirements were not met.

Peripheral blood and bronchoalveolar lavage fluid samples were collected during routine clinical procedures. Briefly, 2 mL of peripheral blood was obtained during standard laboratory testing, and 2 mL of BALF was collected during the bronchoalveolar lavage procedure. No additional invasive procedures or extra blood sampling were performed specifically for this study. All samples used in this research were residual specimens from routine clinical examinations.

In addition to the four paired peripheral blood and BALF samples from MP patients included in the discovery cohort, peripheral blood samples from an additional 13 pediatric MP patients and 20 age-matched healthy children were included as a validation cohort for further immune repertoire analysis. This study was approved by the Ethics Committee of Kunming Children’s Hospital (2023-03-155-K01), and informed consent was obtained from the legal guardians of all participants prior to sample collection.

### PBMC and BALF cell isolation

PBMC were isolated from 2 mL of peripheral blood by density gradient centrifugation using Ficoll-Paque PREMIUM (GE Healthcare, USA) according to the manufacturer’s instructions. Briefly, whole blood was diluted with an equal volume of phosphate-buffered saline (PBS) and carefully layered onto Ficoll solution, followed by centrifugation at 400 × g for 20 min at room temperature. The PBMC layer was carefully collected and washed twice with PBS before further processing.

For BALF, the collected samples were first filtered through a 70-μm cell strainer to remove mucus and debris, and then centrifuged at 400 × g for 10 min at 4 °C to pellet the cells. The cell pellet was resuspended in PBS, and the total number of viable cells was determined by trypan blue staining.

### RNA extraction and library preparation

Total RNA was extracted from both PBMC and BALF-derived cells using TRIzol Reagent (Invitrogen, USA) following the manufacturer’s protocol. The purity and concentration of RNA were assessed using a NanoDrop spectrophotometer (Thermo Fisher Scientific, USA), and RNA integrity was evaluated by agarose gel electrophoresis.

Subsequently, RNA samples that met the quality requirements were shipped on dry ice to Hangzhou ImmuQuad Biotechnology Co., Ltd. (Hangzhou, China) for immune repertoire library construction and sequencing. TCR CDR3 repertoire libraries were prepared using the company’s proprietary ImmuHub^®^ 5’RACE Ultra platform, which employs a 5’ rapid amplification of cDNA ends (5’-RACE) strategy with a patented 5’ adapter ligation approach. This method uses primers designed against the C constant region sequences of TCR chains, allowing for amplification of the complete V(D)J variable region. Importantly, the platform incorporates unique molecular identifiers (UMIs) for each RNA molecule prior to amplification, which enables correction of PCR and sequencing errors and ensures accurate quantification of clonotype abundances. This approach avoids amplification bias associated with multiplex PCR and enables full coverage of the CDR3 region (10, 14). High-throughput sequencing was performed on an Illumina platform with paired-end 150 bp reads (PE150).

### Sequencing quality control

Raw sequencing data generated from the Illumina platform were first subjected to strict quality control. Adapters and low-quality bases were removed using fastp, with the following criteria: (1) reads containing adapter sequences were trimmed; (2) reads with more than 10% of unknown bases (N) were discarded; (3) bases with a Phred quality score < 20 were removed from both ends of the reads; and (4) reads shorter than 50 bp after trimming were excluded from further analysis. Only high-quality clean reads were retained for downstream analysis.

The quality of both raw and clean data was evaluated using FastQC. Parameters including Q20, Q30, GC content, and read length distribution were assessed to determine overall sequencing quality. All samples in this study met the quality control standards, with Q30 values exceeding 90% and no obvious GC bias was observed.

### TCR repertoire assembly and sequence filtering

High-quality clean reads were further processed using MiXCR (version 3.0.13) for TCR repertoire analysis (15). The MiXCR pipeline was used to perform V(D)J gene alignment, clonotype assembly, and CDR3 region extraction based on the IMGT reference database. Only productive (in-frame) TCR rearrangements were retained for downstream analyses.

To ensure the reliability of the repertoire data, several strict filtering criteria were applied: (1) sequences with a CDR3 amino acid length shorter than 5 amino acids were removed; (2) sequences containing ambiguous or abnormal characters, such as “*” or “_”, were excluded; and (3) sequences lacking either a V gene or a J gene annotation were discarded. After filtering, the remaining high-quality and functional TCR CDR3 sequences were used for subsequent clonotype definition, diversity analysis, and comparative repertoire profiling.

### Shared clonotype analysis between paired PBMC and BALF samples

To evaluate the overall similarity of the TCR CDR3 repertoire between paired PBMC and BALF samples from children with MP, a Morisita–Horn index was calculated based on clonotype abundance profiles. This abundance-weighted index reflects both the presence and frequency of shared clonotypes and is less sensitive to differences in sampling depth.

To assess whether the similarity between paired samples was significantly higher than that expected by chance, a permutation-based paired significance test was performed. Specifically, the mean similarity value of the true matched PBMC–BALF pairs was first calculated (“within-pair” similarity). This value was then compared with the distribution of mean similarities obtained from all possible permutations of the four MP labels (k = 4; total of 4! = 24 combinations), generating an exact, one-sided P value to test whether the within-pair similarity was greater than that of randomly paired samples. In addition, clonotype overlap between PBMC and BALF was quantified using two complementary overlap metrics: (1) the proportion of shared clonotypes based on the number of unique clonotype species (species-level overlap), and (2) the abundance-weighted proportion of shared clonotypes based on their cumulative frequencies (abundance-weighted overlap), providing a comprehensive assessment of repertoire overlap between tissues.

Furthermore, scatter plots were generated to visualize the abundance correlation of shared TCR CDR3 clonotypes between PBMC and BALF in each paired sample. Each point in the plot represents a shared clonotype, with its frequency in PBMC plotted against its frequency in BALF. The top 10 most abundant clonotypes in each sample were highlighted in the plots, and their corresponding CDR3 amino acid sequences were annotated for reference.

### V and J gene usage, V–J pairing, CDR3 length and k-mer analysis

The usage patterns of TCR β V and J genes, V–J pairing frequencies, and CDR3 length distributions were analyzed in paired PBMC and BALF samples using the R packages immunarch and VDJtools (16). The frequencies of individual V and J genes as well as V–J combinations were calculated based on productive TCR clonotypes in each sample. All visualizations, including bar plots, heatmaps, and distribution plots, were generated in R (version 4.1.1).

To further assess the similarity of V–J gene usage patterns between samples, cosine similarity analysis was performed on the V–J usage frequency matrices. Briefly, a V–J usage vector was constructed for each sample, and pairwise similarities between samples were calculated using the cosine similarity metric. This analysis was used to quantify the overall similarity of recombination patterns between paired PBMC and BALF samples.

For k-mer analysis, all productive TCR CDR3 amino acid sequences were decomposed into overlapping k-mers using a sliding-window approach with a fixed k value of 5, as implemented in the Immunarch package. The frequency of each k-mer was calculated as the count of that k-mer divided by the total number of k-mers within a given sample. A k-mer × sample frequency matrix was then constructed and used for downstream analyses.

### Antigen annotation of TCR CDR3 sequences

For antigen annotation, the vdjdb.slim.txt file was downloaded from the VDJdb public database (version 2023-06-01). Records corresponding to Homo sapiens and TCR beta were extracted, resulting in 41, 427 entries. The integrated dataset (df0), which contains CDR3 amino acid sequences (CDR3.aa), V/J genes and clone counts, was used for annotation. To avoid mismatches caused by allelic variation, both the VDJdb records and the local datasets were converted to a unified format without allele suffixes (V_short/J_short). An exact-matching strategy based on CDR3 amino acid sequence, V gene and J gene was applied to perform an inner join with the VDJdb human TCR β datasets.

### Statistical analysis

All statistical analyses were performed using R software (version 4.1.1) and GraphPad Prism (version 9.0). Data distribution was first evaluated using the Shapiro–Wilk test. Normally distributed data were analyzed using Student’s t-test, while non-normally distributed data were analyzed using the Wilcoxon signed-rank test or Mann–Whitney U test. For comparisons involving more than two groups, one-way ANOVA or Kruskal–Wallis test was applied, followed by *post hoc* multiple comparison if necessary. Correlations between variables were assessed using Spearman’s rank correlation analysis. Multiple testing correction was performed using the Benjamini–Hochberg false discovery rate (FDR) method where applicable. All statistical tests were two-sided, and a P value < 0.05 was considered statistically significant. Statistical analyses were performed at the appropriate biological level, including patient-level comparisons (n = 4 - 20), sample-level comparisons (n = 8 paired samples), and clonotype-level summaries. Importantly, statistical tests comparing repertoire distributions were performed using sample-level aggregates rather than individual clonotypes to avoid pseudoreplication.

## Results

### Sequencing features of TCR β CDR3 repertoires in paired PBMC and BALF

Productive TCR β CDR3 repertoires were successfully obtained from both PBMC and BALF in all individuals (**Supplementary Table 2**). As expected, PBMC samples exhibited a broad and highly complex circulating T cell pool, resulting in a large number of detectable clonotypes. Notably, despite the relatively low abundance of T cells in the lung compartment, measurable TCR β repertoires were consistently detected in BALF samples, with total clonotype numbers ranging from 1.5 × 10³ to 5.8 × 10^4^ and unique clonotypes ranging from 528 to 5, 286. Although overall clonotype diversity in BALF was lower than that observed in PBMC, the detection of T cell clones in BALF is biologically meaningful, as it reflects the localized immune response within the pulmonary microenvironment at the site of infection.

To investigate clonal expansion patterns in MP patients, we next analyzed the distribution of clonotype frequencies in both PBMC and BALF. Most unique TCR clonotypes in both compartments exhibited varying degrees of clonal expansion(**Supplementary Table 3**). In PBMC, approximately 27.6% of T cells were clonally expanded, whereas this proportion was 50.5% in BALF, indicating distinct clonal architectures between the systemic circulation and the local pulmonary environment.

### TCR CDR3 repertoire overlap between PBMC and BALF in MP patients

The paired study design further enabled the identification of disease-relevant clonotypes shared between the peripheral circulation and the lungs, providing a basis for the subsequent identification of potential M. pneumoniae–associated T cell signatures. The shared T cell clonotypes between PBMC and BALF in each patient were assessed. A total of 45, 88, 76, and 251 shared clonotypes were detected in patients MP1, MP2, MP3, and MP4, respectively (**Supplementary Table 4**). However, among the 472 candidate shared clonotypes, only three were detected in at least two individuals, namely CASSFRGSGANVLTF, CASSLEETQYF, and CASSLGGYEQYF. In addition, 54 clonotypes were commonly detected in the PBMC samples of all four patients, whereas no shared clonotypes were identified in BALF samples across all individuals.

**Figure 2:**
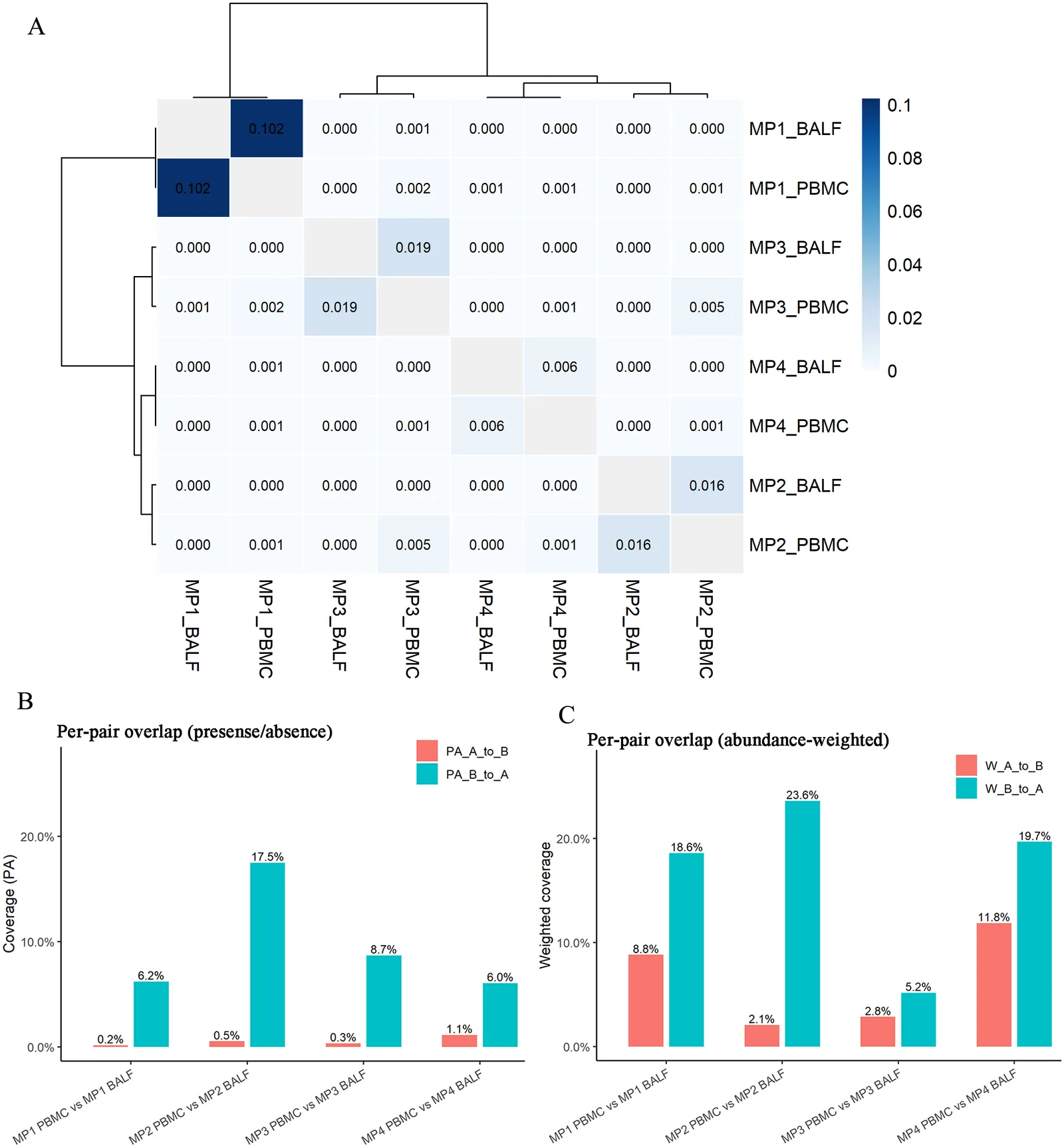
Shared TCR CDR3 repertoire characteristics between paired PBMC and BALF in pediatric MP patients. **(A)** The Morisita–Horn index was used to assess the overall similarity of TCR CDR3 clonotype abundance across eight samples from four MP patients. **(B)** The proportion of shared CDR3 sequences between PBMC and BALF within each individual is shown. **(C)** The contribution of shared clonotypes to the total clonal abundance in PBMC and BALF. MP_PBMC, peripheral blood mononuclear cells from Mycoplasma pneumoniae patients; MP_BALF, bronchoalveolar lavage fluid mononuclear cells from Mycoplasma pneumoniae patients; PA_A_to_B/PA_B_to_A represent the proportion of shared CDR3 sequences in PBMC/BALF, and WA_A_to_B/WA_B_to_A represent the abundance-weighted proportion of shared clonotypes in PBMC/BALF, respectively.

The Morisita–Horn index was used to assess the overall similarity of TCR CDR3 clonotype abundance across the eight samples from four patients (**Figure 2A**). The highest similarity was observed between paired PBMC and BALF samples from the same individual (e.g., approximately 0.10 for MP1), whereas cross-individual comparisons showed consistently low similarity, mostly ranging from 0.00 to 0.02. Importantly, although paired PBMC–BALF samples showed statistically higher similarity than randomly permuted pairings, the absolute Morisita–Horn values remained very low across all comparisons, indicating that the two compartments are still largely distinct in their TCR repertoire composition. An exact global permutation test was conducted to determine whether the similarity between paired PBMC and BALF samples exceeded random expectation. Among 24 possible permutations, only one yielded a mean Morisita–Horn index equal to or greater than the observed value (one-sided p = 0.0417), indicating that the paired structure across the cohort is statistically significant despite the modest absolute similarity.

The presence of shared CDR3 sequences between PBMC and BALF was next quantified within each individual. From a source-based perspective, approximately 0.5% of the CDR3 sequences detected in PBMC were also identified in the corresponding BALF samples, whereas about 9.6% of BALF CDR3 sequences were also detected in matched PBMC samples (**Figure 2B**). This pattern indicates an asymmetric overlap between the two compartments in terms of CDR3 sequence presence. To further assess whether the observed cross-tissue sharing reflected a random distribution, the contribution of shared clonotypes to the total clonal abundance was examined. Although shared clonotypes comprised less than 1% of all unique CDR3 sequences in PBMC, they contributed approximately 8–9% of the total abundance. In BALF, the cumulative abundance of shared clonotypes approached 20% (**Figure 2C**). These data indicate that cross-tissue shared clonotypes are preferentially represented among higher-frequency clones rather than being uniformly distributed across the repertoire.

**Figure 3:**
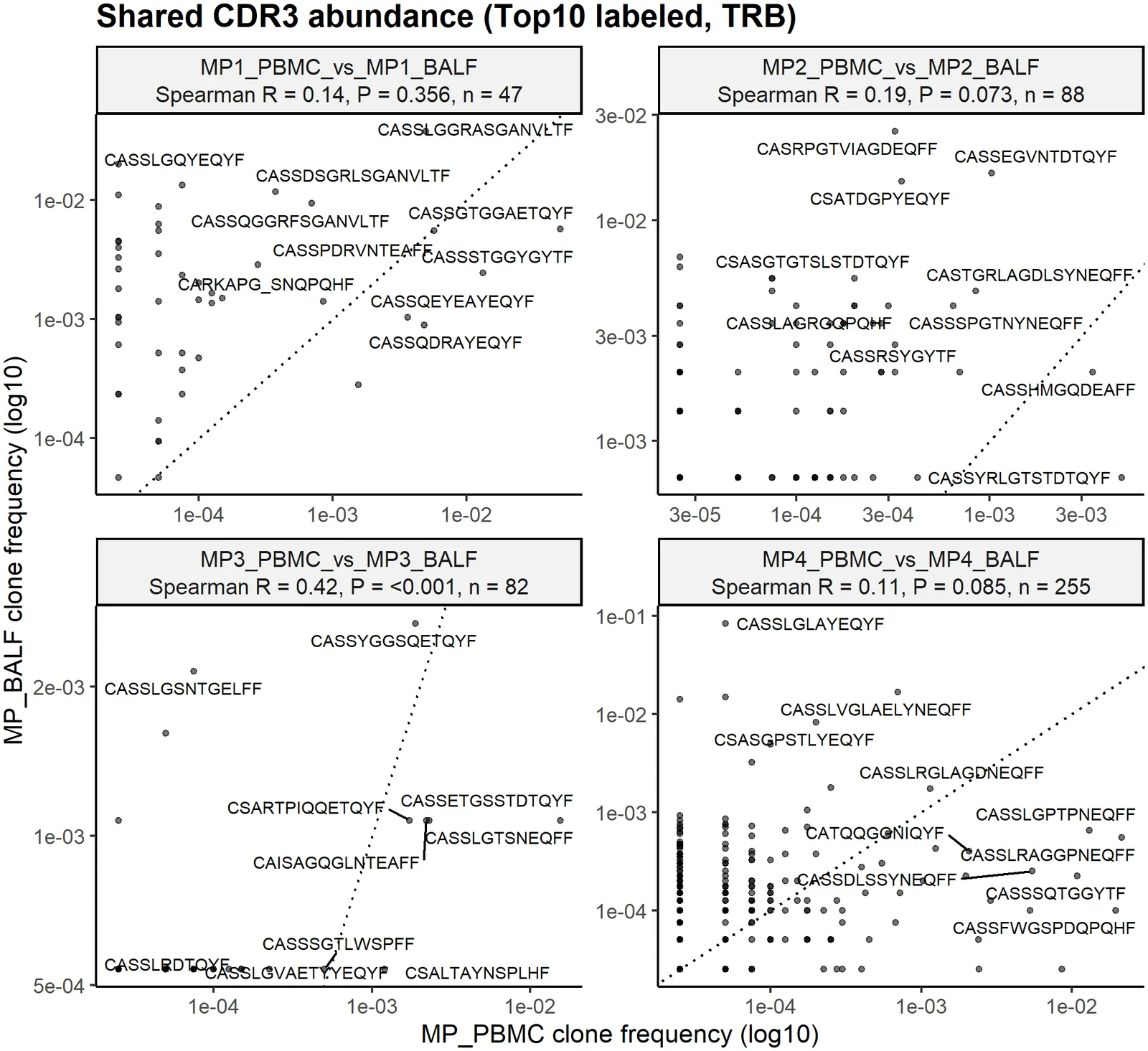
Relative abundance distribution of shared TCRβ CDR3 clonotype abundance between paired PBMC and BALF samples from patients with *Mycoplasma pneumoniae* pneumonia (n = 4 paired samples). MP_PBMC, peripheral blood mononuclear cells from Mycoplasma pneumoniae patients; MP_BALF, bronchoalveolar lavage fluid mononuclear cells from Mycoplasma pneumoniae patients.

The shared CDR3 clonotypes within each paired PBMC–BALF sample were further examined. Most shared clonotypes were distributed within the low- to intermediate-frequency range. The top 10 shared clonotypes in each pair, ranked by geometric mean abundance, were highlighted to illustrate their abundance distribution between paired PBMC and BALF samples (**Figure 3**). The abundance of these shared clonotypes varied considerably among individuals, with a small number of clonotypes contributing disproportionately to the shared repertoire in some patients, whereas such dominant shared clonotypes were scarcely detected in others. These findings further support substantial inter-individual heterogeneity in the abundance patterns of shared clonotypes between PBMC and BALF.

### V and J gene usage of the TCR CDR3 repertoire in paired PBMC and BALF

**Figure 4:**
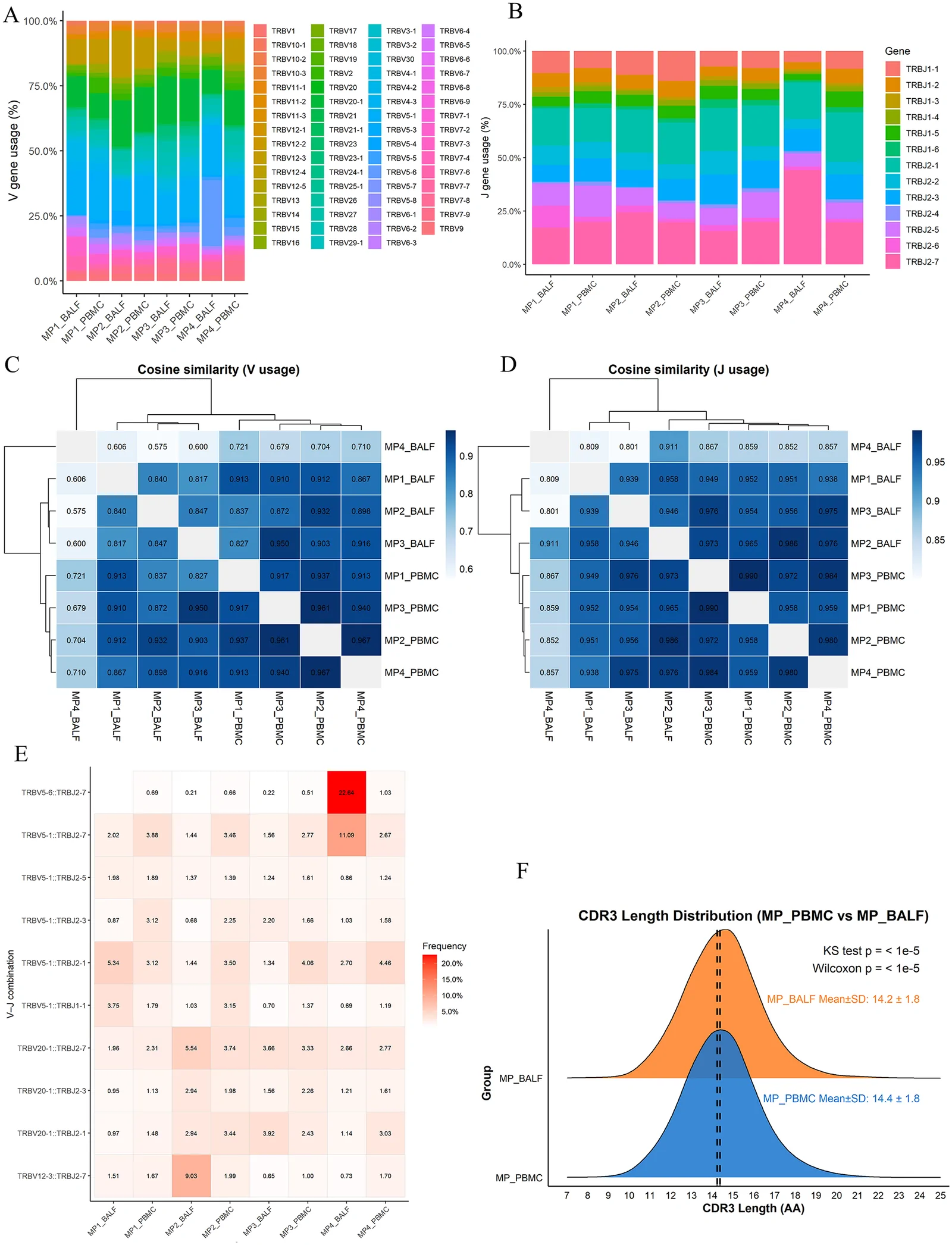
TRB V/J gene usage and CDR3 length distribution in paired PBMC and BALF samples from patients with *Mycoplasma pneumoniae* pneumonia (n = 4 paired samples). **(A)** Overall TRBV gene usage across all samples. **(B)** Overall TRBJ gene usage across all samples. **(C, D)** Cosine similarity analysis based on V and J gene usage. **(E)** Heatmap visualization of the top 10 shared V–J combinations.**(F)** CDR3 length analysis showed a significant difference between PBMC and BALF. MP_PBMC, peripheral blood mononuclear cells from Mycoplasma pneumoniae patients; MP_BALF, bronchoalveolar lavage fluid mononuclear cells from Mycoplasma pneumoniae patients.

In the analysis of TCR V gene usage, an overall stable pattern was observed across all eight samples (**Figure 4A**). TRBV20-1, TRBV5–1 and TRBV12–3 were consistently among the most frequently used V genes in all samples, indicating that they represent dominant V gene segments in the repertoire. Nevertheless, variation was still observed between individuals and between tissue types from the same individual. For example, a slight increase in the relative usage of TRBV5–6 and TRBV28 was noted in BALF samples, whereas higher proportions of TRBV2 and TRBV27 were detected in some PBMC samples.

With respect to J gene usage, TRBJ2–7 was the most frequently used segment in all samples, followed by TRBJ2-3, TRBJ2–5 and TRBJ1-1 (**Figure 4B**). In general, the J gene usage profiles were similar between PBMC and BALF. However, in some individual samples, such as MP4_BALF, the relative abundance of TRBJ2–7 was markedly higher than in other samples.

Cosine similarity analysis based on V gene and J gene usage profiles revealed a tendency for paired PBMC and BALF samples from the same individual to exhibit higher similarity values than samples from different individuals (**Figures 4C, D**). Despite the high diversity of CDR3 sequences and the limited number of shared clonotypes, the overall patterns of V and J gene usage appeared relatively conserved between paired compartments within individual patients.

The VJ pairing patterns were further examined, and several high-frequency VJ combinations were shared between PBMC and BALF. The top 10 shared VJ combinations, ranked by mean relative abundance, were visualized using a heatmap to illustrate their distribution across samples (**Figure 4E**). In most samples, high-frequency combinations associated with TRBV5–1 and TRBV20–1 were consistently detected, including TRBV5-1::TRBJ2-1, TRBV5-1::TRBJ2-3, TRBV5-1::TRBJ2-7, TRBV20-1::TRBJ2-1, TRBV20-1::TRBJ2–3 and TRBV20-1::TRBJ2-7, generally ranging from 0.5% to 5% in individual samples.

### The CDR3 length of TCR CDR3 repertoires in paired PBMC and BALF

A clone-abundance–weighted analysis of the CDR3 amino acid length was performed for T cell clonotypes in PBMC and BALF (**Figure 4F**), and intergroup differences were evaluated using the Kolmogorov–Smirnov (KS) test and the Wilcoxon rank-sum test. A significant difference in the weighted CDR3 length distribution was observed between PBMC and BALF (KS test, p < 1 × 10⁻^5^; Wilcoxon test, p < 1 × 10⁻^5^). Although both compartments retained a dominant peak at approximately 14 amino acids, the distribution in BALF was broader and exhibited an overall rightward shift (**Figure 4F**). Among clonotypes with long CDR3 sequences (≥16 amino acids), recurrent clonotypes accounted for a higher proportion in BALF than in paired PBMC samples (**Supplementary Table 5**). Further abundance-weighted analysis indicated that the extended right tail of the distribution was mainly attributable to high-frequency clonotypes in BALF. In contrast, this feature was less pronounced in PBMC, indicating a compartment-specific difference in CDR3 length distribution.

### Database annotation and k-mer profiling

**Figure 5:**
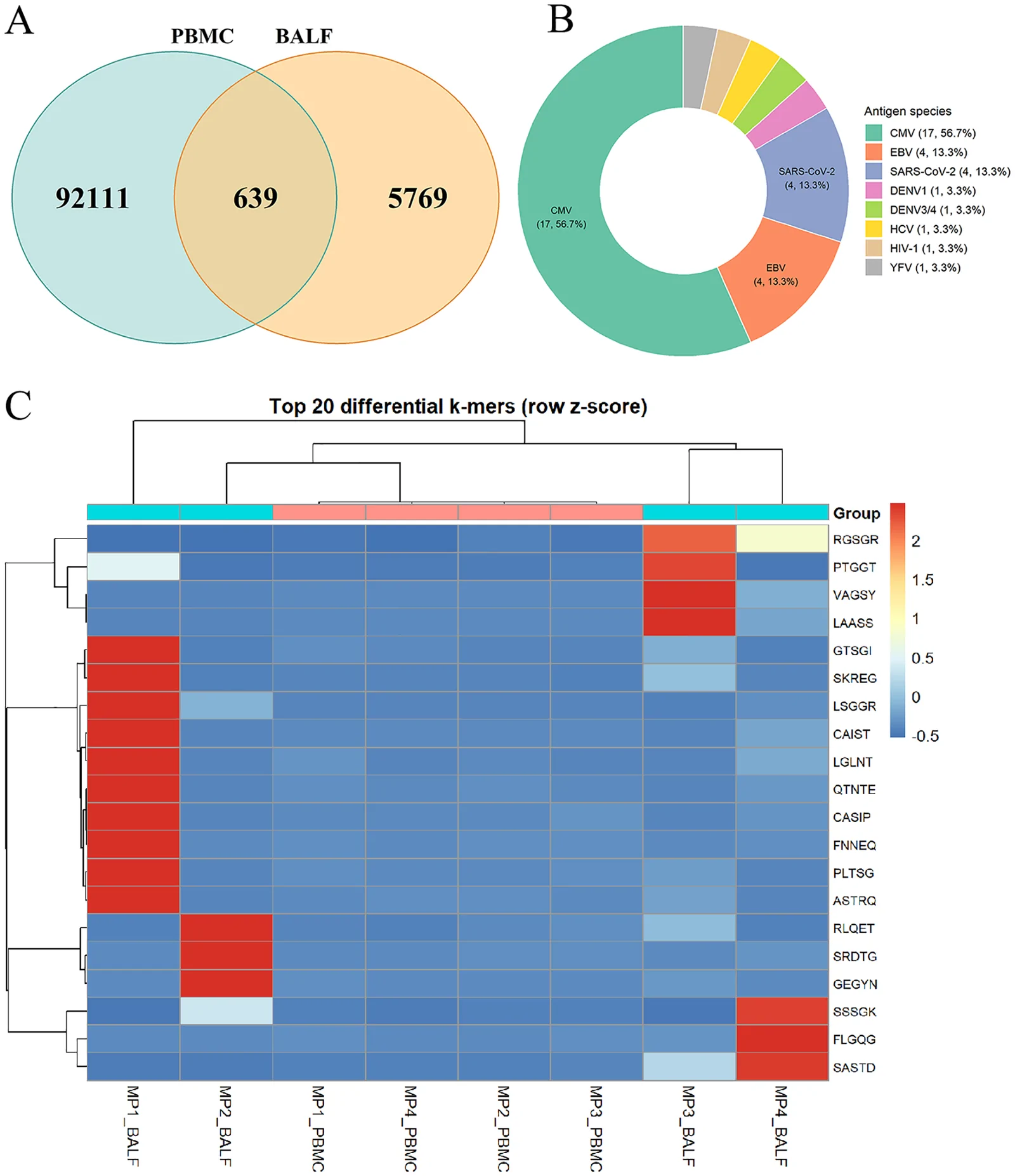
Antigen annotation and k-mer features of shared TCR CDR3 sequences in paired PBMC and BALF samples from patients with *Mycoplasma pneumoniae* pneumonia (n = 4 paired samples). **(A)** Number of shared CDR3 sequences between paired PBMC and BALF samples. **(B)** Antigen annotation of shared TCRβ CDR3 sequences based on the VDJdb database. **(C)** Heatmap and hierarchical clustering of the top 20 differential k-mers (k = 5) based on row z-scores. PBMC, peripheral blood mononuclear cell; BALF, bronchoalveolar lavage fluid.

To explore whether the clonotypes shared between PBMC and BALF included previously reported antigen-specific sequences, we performed a database-based annotation analysis. A total of 639 shared CDR3 sequences were identified between PBMC and BALF samples (**Figure 5A**), corresponding to 1, 038 unique CDR3+V+J combinations. Among these shared combinations, 31 (approximately 3.0%) matched previously reported, antigen-annotated TCR CDR3 sequences in the VDJdb database, representing 25 unique CDR3 amino acid sequences. These annotated shared clonotypes were predominantly associated with viral antigens, with cytomegalovirus (CMV) being the most frequent, including several classical pp65 epitope–specific TCRs. In addition, clonotypes specific for Epstein–Barr virus (EBV) and SARS-CoV-2 were identified, along with a small number associated with HIV-1, hepatitis C virus, dengue virus and yellow fever vaccine–related epitopes (**Figure 5B**).

To further characterize the sequence-level differences between PBMC and BALF TCRβ repertoires, we analyzed the top 20 differential k-mers based on row z-scores. Hierarchical clustering of these k-mers revealed a clear separation between PBMC and BALF samples, indicating distinct sequence composition patterns in circulating and lung T cell populations (**Figure 5C**). Several k-mers, including GTSGI, SKREG, LSGGR and CAIST, were preferentially enriched in BALF samples, whereas others such as SSSGK, FLGQG and SASTD showed higher relative abundance in specific individuals. These results suggest that the local pulmonary TCR CDR3 repertoire in MP-infected children exhibits unique sequence motifs that are distinct from those observed in peripheral blood.

### Validation of paired TCR CDR3 repertoire features in expanded pediatric MP and healthy cohorts

**Figure 6:**
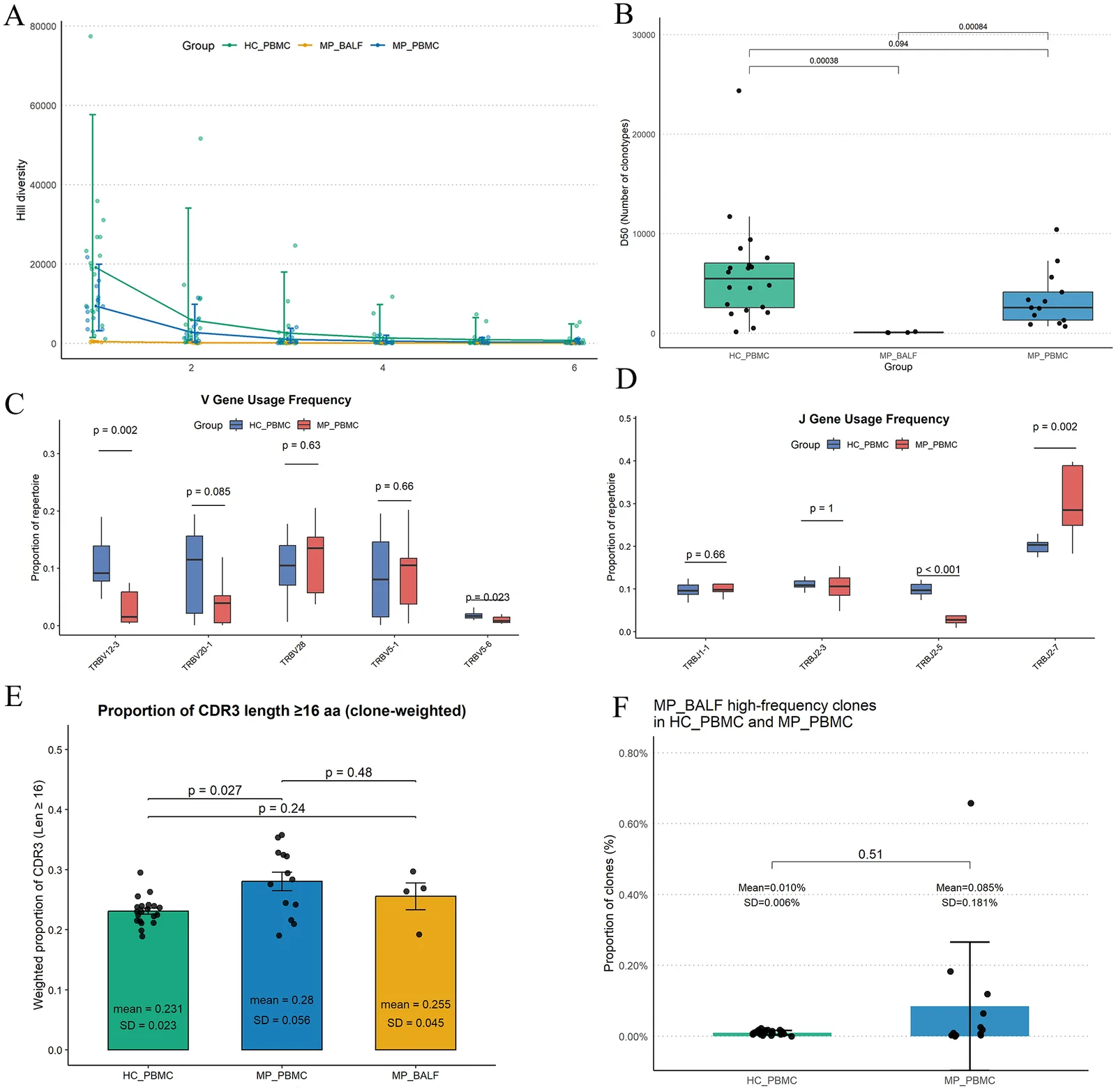
Validation of TCR CDR3 repertoire alterations in an expanded cohort of pediatric MP patients and healthy controls **(A)** Hill diversity analysis of TCR CDR3 repertoires in HC_PBMC, MP_PBMC, and MP_BALF samples. **(B)** Comparison of D50 values (number of clonotypes accounting for 50% of total reads) among the three groups. **(C)** Differential usage of five high-frequency TRBV genes identified from paired-sample analysis in HC_PBMC and MP_PBMC. **(D)** Differential usage of four selected TRBJ genes between HC_PBMC and MP_PBMC. **(E)** Proportion of CDR3 sequences ≥16 amino acids in HC_PBMC, MP_PBMC, and MP_BALF. **(F)** Cumulative frequency of the top 20 expanded BALF clonotypes in HC_PBMC and MP_PBMC. HC_PBMC, peripheral blood mononuclear cells from healthy controls (n = 20); MP_PBMC, peripheral blood mononuclear cells from *Mycoplasma pneumoniae* patients (n = 13); MP_BALF, bronchoalveolar lavage fluid mononuclear cells from *Mycoplasma pneumoniae* patients (n = 4). For analyses involving multiple comparisons (**Figure 5C**), p-values were adjusted using the Benjamini–Hochberg false discovery rate (FDR) method. All other statistical comparisons were based on unadjusted p-values.

To further validate the findings derived from the paired PBMC–BALF analysis, we extended the repertoire comparison to an independent pediatric cohort consisting of 13 children with MP infection and 20 healthy children (HC). Hill diversity analysis revealed the lowest diversity in MP_BALF, followed by MP_PBMC, while HC_PBMC exhibited the highest diversity (**Figure 6A**). Consistently, the D50 (number of clonotypes accounting for 50% of total reads) showed a similar pattern, with the MP_BALF group displaying significantly reduced clonotype richness compared with both MP_PBMC and HC_PBMC (**Figure 6B**).

Based on the five high-frequency TRBV genes identified from the paired BALF–PBMC analysis, their usage was further compared between HC_PBMC and MP_PBMC. TRBV12–3 and TRBV5–6 were significantly downregulated in MP_PBMC, while TRBV20–1 also showed a decreasing trend (p = 0.085), and no significant differences were observed for the remaining two TRBV genes (**Figure 6C**).

Similarly, among the four selected TRBJ genes, TRBJ2–5 was significantly less frequently used in MP_PBMC than in HC_PBMC, whereas TRBJ2–7 showed significantly lower usage in HC_PBMC compared with MP_PBMC (**Figure 6D**).

In line with the paired-sample observation that BALF exhibited a broader distribution and overall rightward shift in CDR3 length, with an increased proportion of long CDR3 sequences (≥16 amino acids), we further quantified long CDR3 frequencies in the expanded cohorts. The proportion of CDR3 ≥16 amino acids was significantly higher in MP_PBMC compared with HC_PBMC (**Figure 6E**).

Finally, the top 20 recurrent clonotypes identified in each BALF sample were traced in the PBMC repertoires. Although no statistically significant difference was observed between the two groups, the average frequency of these BALF-recurrent clonotypes was higher in MP_PBMC (0.085%) than in HC_PBMC (0.010%) (**Figure 6F**), suggesting a trend toward enhanced peripheral expansion of BALF-associated clonotypes in MP patients.

## Discussion

In this study, we systematically characterized the TCR β CDR3 repertoire in paired PBMC and BALF from children with MP pneumonia, and further validated these findings in an expanded cohort including additional MP patients and healthy controls. Our results reveal pronounced compartment-specific and disease-associated changes in the TCR repertoire, providing new insight into the local and systemic T cell immune responses in pediatric MP infection.

Bronchoalveolar lavage fluid offers a direct and reliable window into the immune microenvironment of the lower respiratory tract and has been widely used to investigate the local host response in pulmonary diseases, including pediatric MP (17–19). Unlike peripheral blood, which mainly reflects systemic immune surveillance, BALF captures immune cells and molecular events occurring at the site of infection and inflammation (20). In children with MP, BALF-based studies have demonstrated that disease severity is more closely associated with host immune activation—particularly innate and adaptive immune responses—than with microbial burden alone (21). Alterations in BALF cellular composition, including increased lymphocyte proportions and changes in macrophage and neutrophil distributions, have been linked to complications, extrapulmonary manifestations, and refractory disease (22, 23). Transcriptomic and single-cell analyses further revealed enrichment of immune-related pathways in BALF, such as T cell receptor signaling, NK cell–mediated cytotoxicity, and monocyte/macrophage activation, while severe cases exhibited exhausted T cells and inflammatory macrophage phenotypes, suggesting persistent antigenic stimulation and dysregulated T cell function in the lung microenvironment (24). Consistent with these observations, our TCR repertoire analysis demonstrated that MP_BALF was characterized by markedly reduced diversity, a higher proportion of recurrent clonotypes, and a distinct right-shifted CDR3 length distribution with enrichment of long CDR3 sequences (≥16 amino acids), suggesting a pattern consistent with localized clonal expansion within the infected lung microenvironment, potentially influenced by antigen exposure and/or inflammatory cues, although direct evidence for *Mycoplasma pneumoniae*–specific TCR recognition remains limited.

Although most TCR clonotypes identified in our study were not shared between PBMC and BALF, a proportion of shared clonotypes was still detectable in paired samples, and V and J gene usage patterns were more consistent between the two compartments within the same patient than across different individuals. These observations suggest that a subset of circulating peripheral T cells may migrate into lung tissue under infectious or inflammatory conditions and undergo local expansion driven by local inflammatory cues and/or antigen exposure, rather than direct evidence of defined *Mycoplasma pneumoniae*–specific recognition (25, 26). In addition, clonotypes preferentially enriched and expanded in BALF also exhibited relatively higher abundances in MP-PBMC, further supporting a dynamic link between focal pulmonary immune responses and the peripheral immune system (27, 28). Unfortunately, only a small fraction of the shared clonotypes matched previously reported pathogen-specific TCR sequences in public databases, and most of which were associated with viral antigens. This is likely attributable to the limited availability of Mycoplasma pneumoniae–related TCR information in current databases, highlighting the importance of establishing a dedicated TCR resource for pediatric respiratory infections.

In the validation analysis of the expanded cohort, we further confirmed a general reduction in TCR diversity accompanied by an increase in CDR3 length in children with MP infection, suggesting that children with MP infection exhibit a repertoire pattern characterized by increased representation of recurrent clonotypes. Although such an over-focused clonal expansion may facilitate a stronger adaptive immune response against specific antigens, it may also restrict the breadth of immune recognition and be accompanied by amplified local inflammatory responses, thereby contributing to the development and progression of lung tissue injury. This feature is consistent with observations reported in various infectious and autoimmune diseases (29, 30). In addition, the increased CDR3 length observed in our study may reflect altered repertoire selection patterns during infection, potentially affecting antigen recognition properties (31). However, the functional consequences of these changes require further investigation.

Similar patterns of reduced repertoire diversity and increased clonotype expansion have also been reported in other infectious and immune-mediated diseases, including viral respiratory infections, autoimmune disorders, and inflammatory conditions, where persistent immune activation or antigen exposure contributes to T-cell clonal remodeling (32–34). These observations suggest that although the specific antigenic drivers may differ among diseases, adaptive immune repertoire remodeling represents a common feature of immune responses under sustained inflammatory stress.

The usage of V and J genes plays a critical role in shaping the TCR repertoire and determining its antigen recognition potential (35, 36). In this study, several high-frequency VJ combinations were consistently shared between paired PBMC and BALF samples, reflecting both compartmental conservation and the presence of dominant clonotypes. However, marked inter-individual variability was observed, which may reflect differences in each individual’s MHC repertoire, leading to selective expansion of T cells recognizing distinct antigenic peptides presented by their unique MHC molecules (37, 38). Validation in the expanded cohort confirmed that TRBV12–3 and TRBV5–6 were significantly downregulated in MP_PBMC compared with healthy controls, while TRBV20–1 also showed a decreasing trend, and among the J genes, TRBJ2–5 was reduced and TRBJ2–7 was relatively increased in MP_PBMC. These findings indicate that children with MP pneumonia exhibit biased VJ gene usage, which may reflect infection-associated immune activation and repertoire remodeling.

Several limitations of this study should be acknowledged. First, the cross-sectional design precludes distinguishing whether the observed TCR features are infection-induced or pre-existing, and thus our findings are correlative rather than causal. Second, the sample size was relatively small, particularly for the paired BALF–PBMC cohort (n = 4). Because BALF samples are difficult to obtain from pediatric patients, the present study should be considered exploratory and hypothesis-generating in nature. Although several compartment-associated TCR repertoire features were identified, these findings remain to be confirmed in larger independent cohorts. Third, this study focused primarily on bulk TCR β-chain repertoire sequencing and did not include T-cell subset characterization, flow cytometric validation, or sorted T-cell population analyses. Therefore, the contribution of specific T-cell subsets (e.g., CD4^+^, CD8^+^, or memory/effector populations) to the observed repertoire differences could not be directly determined. In addition, the lack of single-cell transcriptomic profiling limited the ability to link clonotype expansion with functional phenotypes or activation states. Future investigations combining TCR sequencing with high-dimensional phenotyping (flow cytometry or single-cell multi-omics), functional assays, and clinical metadata will be necessary to fully elucidate the cellular origin and functional significance of the recurrent clones. Such integrated approaches may also help determine whether repertoire features identified in the present study can serve as clinically useful biomarkers for disease stratification or therapeutic monitoring.

## Data Availability

The processed TCR β CDR3 sequencing data and analysis scripts generated and analyzed during this study are publicly available in the Zenodo repository (Accession No. 18028866) at https://doi.org/10.5281/zenodo.18028866.
